# Risk factors for automobile accidents caused by falling asleep while driving in obstructive sleep apnea syndrome

**DOI:** 10.1007/s11325-015-1145-7

**Published:** 2015-02-26

**Authors:** Aki Arita, Ryujiro Sasanabe, Rika Hasegawa, Atsuhiko Nomura, Reiko Hori, Mamiko Mano, Noriyuki Konishi, Toshiaki Shiomi

**Affiliations:** Department of Sleep Medicine, Aichi Medical University School of Medicine, 1-1 Yazakokarimata, Nagakute, Aichi 480-1195 Japan

**Keywords:** Automobile accidents, Obstructive sleep apnea, Epworth sleepiness sale, Falling asleep, Risk factors

## Abstract

**Purpose:**

We examined the risk factors for automobile accidents caused by falling asleep while driving in subjects with obstructive sleep apnea syndrome (OSAS).

**Methods:**

We asked licensed drivers with history of snoring and excessive daytime sleepiness who had undergone polysomnography (PSG) at the Department of Sleep Medicine/Sleep Disorders Center at Aichi Medical University Hospital to complete the questionnaires on accidents caused by falling asleep while driving. As a subjective measure of sleepiness, we used the Epworth sleepiness scale (ESS). Based on PSG results, 2387 subjects diagnosed with OSAS were divided into three groups according to apnea-hypopnea index (AHI): mild-to-moderate (5 ≤ AHI < 30), severe (30 ≤ AHI < 60), and very severe (AHI ≥ 60). We performed univariate and multivariate logistic regression on variables that might explain falling asleep at the wheel.

**Results:**

We compared results between each group and simple snorers (394 subjects with AHI < 5) and found the group with very severe OSAS reported significantly higher rates of driving when drowsy and having accidents in the past 5 years due to falling asleep.

**Conclusions:**

Our multivariate analysis suggests that scores on the ESS and patient-reported frequency of feeling drowsy while regular driving and working are related to automobile accidents caused by falling asleep while driving.

## Introduction

The majority of people with obstructive sleep apnea syndrome (OSAS) also have lifestyle-related diseases that are complications of obesity, hypertension, type 2 diabetes, and metabolic syndrome [1–4]. Even though symptoms of hypersomnia in OSAS patients are secondary to OSAS (i.e., arousal reactions during nocturnal sleep due to frequent respiratory arrest that also causes sleep to be lighter and more fragmented, resulting in symptoms of hypersomnia), OSAS patients report higher frequencies of feeling drowsy while driving [5], and the extent of damage caused by accidents due to falling asleep while driving is greater than that from other driving accidents. Accordingly, accidents caused by falling asleep while driving tend to result in serious injury or fatalities [6]. However, symptoms of hypersomnia do not necessarily correlate with the apnea-hypopnea index (AHI) [7], and previous research has not fully elucidated the factors associated with automobile accidents due to drowsiness and OSAS. Thus, we examined potential risk factors for automobile accidents caused by falling asleep while driving.

## Methods

### Subjects

Subjects in this study were 2387 licensed drivers who presented with primary complaints of snoring and excessive daytime sleepiness (EDS) and diagnosed with OSAS after undergoing polysomnography (PSG) at Aichi Medical University Hospital between 2000 and 2011. Subjects were diagnosed with OSAS based on the definition of the American Academy of Sleep Medicine (AASM) [8]. Subjects with sleep disorders other than OSAS observed during PSG were excluded from the study. Smoking habit and drinking habit were confirmed by questionnaires. At the first medical examination, the medical histories about arteriosclerosis, hypertension, diabetes, and cardiovascular disease were extracted from their medical records.

In this study, we divided subjects into three groups based on AHI severity level: mild-to-moderate (5 ≤ AHI < 30), severe (30 ≤ AHI < 60), and very severe (AHI ≥ 60). For comparison, we included simple snorers, which consisted of 394 subjects with primary complaints of snoring or sleepiness who underwent PSG at Aichi Medical University Hospital, had AHI < 5, and were not diagnosed with other sleep disorders and hypersomnia. Subjects with any of the following information in their medical record were excluded from the study: (1) those observed to have idiopathic hypersomnia and narcolepsy on the multiple sleep latency test, (2) depression, (3) receiving treatment for schizophrenia, (4) taking sedative drugs, or (5) scoring less than 26 points on the mini-mental state examination [9], which would suggest the possibility of declining cognitive function.

### Polysomnography

Nocturnal PSG was performed with multichannel monitoring including neurophysiological variables (electroencephalogram, electrooculogram, chin electromyogram) and cardio-respiratory variables (chest well motion, nasal pressure, arterial oxygen saturation, and electrocardiogram). Continuous recordings were obtained with a computerized diagnostic system (P series™: Compumedics, Melbourne, Australia; or Alice 4™: Respironics Inc., Pittsburgh, USA). The sleep record was analyzed manually according to the criteria of Rechtshaffen and Kales using a 30-s epoch [10].

As scoring of respiratory events, apnea was defined as a cessation of airflow for at least 10 s. Central apneas were identified by the absence of respiratory effort during cessation of airflow. Hypopnea was defined as a 50 % reduction in airflow and/or respiratory effort, accompanied by oxygen desaturation of more than 3 %, despite of following arousal. AHI was defined as the hourly number of episodes of apnea and hypopnea combined and was calculated as an indicator of OSAS severity [8]. Three and 4 % oxygen desaturation index (3 and 4%ODI) were extracted supplementarily. ODI was defined as the hourly the number of the blood’s oxygen level drops by 3 % (or 4 %) and more from baseline. Furthermore, arousal index was defined as the hourly number of arousal that lasts at least 3 s during sleep and was extracted as an independent factor.

### Measures of sleepiness

As a subjective measure of sleepiness, we used the Epworth sleepiness scale (ESS) by Johns [11].

When licensed drivers underwent PSG, we asked them to complete a questionnaire on accidents caused by falling asleep while driving. The questionnaire consisted of five questions on the following: (1) whether they were employed in irregular shift work or not, (2) driving distances per month (shorter than 1000 km, between 1000 and 2000 km, between 2000 and 5000 km, or longer than 5000 km), (3) the frequency of feeling drowsy during regular driving and working (frequent, sometimes, rarely, or never), (4) having experienced falling asleep while driving, and (5) having experienced automobile accidents in the past 5 years due to falling asleep while driving. Data on driving distance per month and frequency of feeling drowsy during regular driving and working were divided into two groups around the median.

### Statistical analysis

Continuous variables are shown as the mean ± standard deviation, and categorical variables are shown as frequencies and percentages. The Welch *t* test and Wilcoxon test were performed on continuous variables, and the chi-square test was performed on categorical variables for the comparison between simple snorers with AHI < 5 and the OSAS group with AHI ≥ 5. For comparison of the accident rate associated with drowsy driving, the chi-square test was performed for each of the three groups of subjects with OSAS (mild-to-moderate, severe, and very severe). To assess factors for accidents caused by falling asleep at the wheel, the dependent variable was whether the subject reported having an accident due to falling asleep at the wheel during the past 5 years and independent variables were age, AHI, 3 % oxygen desaturation index (ODI), 4%ODI, ESS, body mass index (BMI), smoker, alcoholic use, sleep latency, minimum SpO_2_, arousal index, leg movement index, the presence of irregular shift work, driving distance per month (less than 1000 km or greater than 1000 km), and the frequency of feeling drowsy during regular driving and working (more than sometimes or less than rarely). We performed univariate logistic regression and multivariate logistic regression on those variables. Since the ESS value was a factor for accidents due to drowsy driving as a result of multivariate logistic regression, we examined the cutoff value for the ESS from the positive receiver operating characteristic curve (ROC curve) for preventing accidents due to drowsy driving. JMP and SPSS software were used for all statistical analysis, and critical *p* values were <0.05.

## Results

### Comparison between the three AHI severity groups and simple snorers

Subjects were 2387 subjects diagnosed with OSAS (AHI ≥ 5) (2171 male and 216 female) and 394 subjects diagnosed as simple snorers (AHI < 5) (303 male and 91 female) by PSG. As described above, the 2387 subjects with OSAS who had an AHI ≥ 5 were divided into three groups based on AHI severity. The accident rates for falling asleep while driving in the severe and very severe group were 1.5 and 2.6 times higher than the rate for the simple snorers (9.8 % [77/790] in the severe group and 16.9 % [82/484] in the very severe group vs. 6.4 % [25/394] in the simple snorers; *p* < 0.05, 0.01, respectively) (Table 1).

**Table 1:** Comparison of OSAS subject and simple snorers

	Simple snorer AHI < 5 *N* = 394 M: 3030, F: 91	Mild/moderate OSAS 5≦AHI < 30 *N* = 1113 M: 964, F: 149	Severe OSAS 30≦AHI < 60 *N* = 790 M: 745, F: 36	Very severe OSAS AHI≧60 *N* = 484 M: 453, F: 31	All OSAS AHI≧5 *N* = 2387 M: 2171, F: 216
Age (years)	44.9 ± 13.7	50.9 ± 12.9**	52.0 ± 12.5**	45.7 ± 11.7	50.2 ± 12.8**
AHI (/h)	2.1 ± 1.5	15.6 ± 7.1**	44.2 ± 8.9**	76.1 ± 14.5**	37.3 ± 25.2**
Epworth sleeping scale	8.6 ± 4.9	9.5 ± 5.0**	9.7 ± 5.1**	11.6 ± 5.2**	10.0 ± 5.1**
BMI (kg/m^2^)	24.0 ± 4.2	25.3 ± 3.8**	26.9 ± 4.3**	32.0 ± 5.9**	27.2 ± 5.1**
Hypertension (%)	20.4	34.9**	46.3**	38.9**	38.6**
Diabetes (%)	4.7	11.1**	12.1**	16.7**	12.3**
Cardiovascular disease (%)	4.7	8.8	10.0*	4.6	8.5
Arteriosclerosis (%)	1.4	3.3	3.7	0.93	3.0
Smoker (%)	22.8	18.9	23.6	33.3*	22.5
Alcoholic use (%)	37.4	43.9	47.4*	35.2	43.5
Sleep latency (minutes)	20.8 ± 22.6	12.4 ± 15.8**	11.7 ± 14.9**	9.9 ± 17.1**	11.7 ± 15.8**
Minimum SpO_2_ (%)	89.4 ± 7.2	83.2 ± 7.4**	75.1 ± 10.5**	65.7 ± 12.6**	77.0 ± 11.8**
3%ODI (/h)	1.9 ± 1.8	13.0 ± 7.4**	38.6 ± 10.6**	71.7 ± 12.8**	34.3 ± 24.9**
4%ODI (/h)	1.6 ± 1.6	11.8 ± 7.7**	34.6 ± 12.8**	71.3 ± 15.1**	24.9 ± 24.0**
Arousal index (/h)	15.5 ± 9.9	23.2 ± 10.5**	42.4 ± 11.8**	70.4 ± 16.6**	39.2 ± 21.8**
Leg movement index (/h)	1.5 ± 3.1	6.6 ± 17.8**	3.8 ± 11.2	1.1 ± 4.8	4.7 ± 14.3**
Habitual sleep time (h)	6.8 ± 1.2	7.0 ± 3.8	6.8 ± 1.1	6.8 ± 1.1	6.9 ± 2.9
Irregular shift worker; (%)	14.6	13.2	11.9	15.5	13.3
Driving distance/month; longer than 1000 km; (%)	34.5	37.8	43.5**	54.3**	43.0**
Frequency of feeling drowsy during regular driving regular driving and working; more than sometimes;(%)	44.9	47.6	51.2*	65.7**	52.4**
Experience of drowsy driving; (%)	25.1	30.0	33.3*	51.6**	35.3**
Automobile accidents in the past 5 years; (%)	6.4	9.1	9.8*	16.9**	10.9**

Continuous variables are shown as average ± SD, and categorical variables are shown as percentages. Driving distance/month and the frequency of feeding drowsy during regular driving and working were divided into two groups around the median

*OSAS* obstructive sleep apnea syndrome, *AHI* apnea-hypopnea index, *BMI* body mass index, *ODI* oxygen desaturation index

*p < 0.05, **p < 0.01 vs. simple snorers

The mild-to-moderate and severe groups were significantly older than simple snorers. Significant differences also were found between all groups for each of the following factors: ESS, BMI, hypertension, diabetes, arousal index sleep latency, and minimum SpO_2_. When compared with simple snorers, the three OSAS groups showed significantly higher ESS, BMI, hypertension, diabetes, arousal index values, 3%ODI and 4%ODI, and lower values for sleep latency and minimum SpO_2_.

The severe group had significantly greater rates of cardiovascular disease and alcohol use compared with simple snorers. The very severe group had a significantly higher percentage of smokers compared with simple snorers. The mild-to-moderate group showed more leg movements when compared with simple snorers.

The severe and very severe groups showed significantly higher values than simple snorers for the following: driving distance per month (further than 1000 km), feeling drowsy more than sometimes during regular driving and working, having experienced drowsy driving, and having experienced automobile accidents in the past 5 years due to falling asleep while driving. There were no significant differences in arteriosclerosis, habitual sleep time, and percentage of subjects with irregular shift work (Table 1).

### Risk factors for automobile accidents caused by falling asleep while driving

**Fig. 1:**
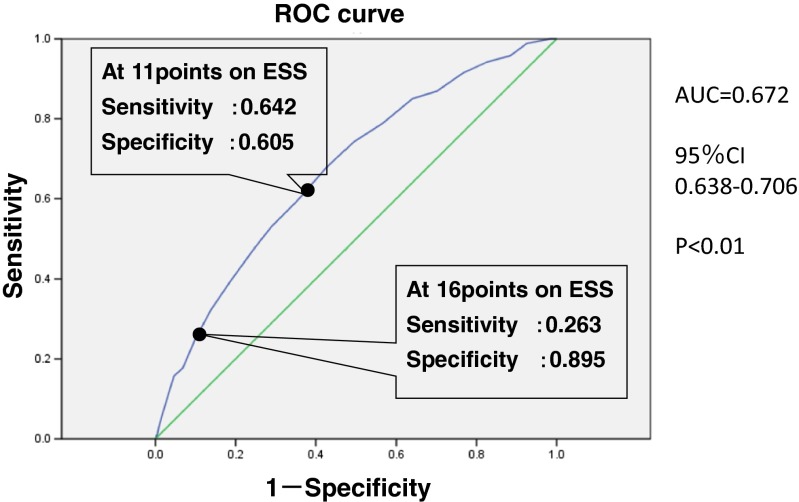
ROC curve for the Epworth sleepiness scale. *ROC* receiver operating characteristic, *AUC* area under the ROC curve, *CI* confidence interval

The ESS and “frequency of feeling drowsy during regular driving and working” were identified as risk factors for automobile accidents caused by falling asleep while driving. Furthermore, an ESS cutoff value of 16 points seems an appropriate value for evaluating the risk of automobile accidents caused by falling asleep while driving. We performed a univariate logistic regression to identify factors associated with automobile accidents caused by falling asleep while driving. Significant factors were age, AHI, ESS, BMI, sleep latency, minimum SpO_2_, arousal index, 3%ODI, 4%ODI, driving farther than 1000 km/month, and feeling drowsy more than sometimes during regular driving and working (Table 2). There were no significant differences in smoker, alcoholic use, leg movement index, habitual sleep time, and percentage of people with irregular shift work. In a multivariate logistic regression using those variables as independent variables, we identified ESS and feeling drowsy more than sometimes during regular driving and working as risk factors for automobile accidents due to falling asleep while driving. Because the correlation between the AHI, the arousal index, 3%ODI, and 4%ODI was high, we performed a multivariate analysis to consider the influence of each factors. Other factors showed no correlation or interaction. For those subjects who reported having accidents due to driving while drowsy, the receiver operating characteristics (ROC) curve for ESS was significant and indicated fair accuracy (AUC 0.672: 95 % CI: 0.638–0.706; *p* < 0.01). At 11 points on the ESS, specificity was 0.605 and sensitivity was 0.642. At 16 points on ESS, specificity was 0.895 and sensitivity was 0.263 (Fig. 1).

**Table 2:** Risk factors for automobile accidents caused by falling asleep while driving OSAS patients

	Univariate				Multivariate logistic regression^a^	
	OR	95%CI	*P*	OR	95%CI	*P*
Age (years old)	0.99	0.98–1.00	<0.01			NS
AHI (/h)	1.01	1.00–1.02	<0.01	1.08	1.048–1.114	NS
Epworth sleepiness scale	1.13	1.10–1.16	<0.01			<0.01
BMI (kg/m^2^)	1.05	1.02–1.07	<0.01			NS
Smoker			NS			
Alcoholic use			NS			
Sleep latency (min)	0.98	0.97–0.99	<0.01			NS
Minimum SpO_2_	0.98	0.97–0.99	<0.01			NS
3%ODI (/h)	1.01	1.01–1.02	<0.01			
4%ODI (/h)	1.01	1.00–1.02	<0.01			
Arousal index (/h)	1.01	1.01–1.02	<0.01			
Leg movement index (/h)			NS			
Habitual sleep time (/h)			NS			
Irregular shift worker			NS			
Driving distance/month; longer than 1000 km	1.76	1.35–2.28	<0.01			NS
Frequency of feeling drowsiness during regular driving and working; more than sometimes	3.31	2.45–4.48	<0.01	1.98	1.40–2.81	<0.01
Gender			NS			

Driving distance/month and the frequency of feeling drowsy during regular driving and working were divided into two groups around the median. We performed multivariable analysis by the combinations of the following four patterns (set1 + AHI, set1 + arousal index, set1 + 3%ODI, set1 + 4%ODI). Set1 included age, Epworth sleepiness scale, BMI, sleep latency, minimum SpO_2_, driving distance/month: longer than 1000 km and frequency of feeling drowsiness during regular driving and working, more than sometimes

*ODI* oxygen desaturation index, *OSAS* obstructive sleep apnea syndrome, *OR* odds ratio, *CI* confidence interval, *AHI* apnea-hypopnea index, *BMI* body mass index

^a^Because the AHI strongly correlated with arousal index (*r* = 0.881), 3%ODI (*r* = 0.966), and 4%ODI (*r* = 0.943), multivariable analysis were performed these factor separately

## Discussion

Many studies have examined the danger of drowsy driving in OSAS patients and the correlation between OSAS and accidents caused by driving while drowsy. Studies have reported that patients with OSAS are at greater risk for experiencing drowsy driving [5] and risks are higher in groups with severe AHI compared with those with mild or moderate AHI [12–15]. Reports also have described a direct relationship between driving performance and OSAS severity (i.e., people with more severe OSAS showed worse driving performance) [16].

In our study, many subjects in the severe group (AHI ≥ 30/h) complained about frequent drowsiness during regular driving or working. Additionally, the number of subjects in this group who had experienced drowsy driving and had automobile accidents caused by falling asleep while driving in the past 5 years was significantly higher. Further, these tendencies were more remarkable in subjects in the very severe AHI group (AHI ≥ 60/h). In a report that addressed arrhythmia and sleep apnea severity, the frequency of awakening with hypoxia and then sleeping again influenced the presence of heart vascular disorders [17]. Therefore, we classified AHI ≥ 60/h as the very severe group and AHI ≥ 30/h as the severe group and compared the accident rate. In an investigation of the effect of sleepiness on driving performance, subjects were most at risk of diverting from the main lane and driving much slower while under the stress of sleep deprivation [18]. Various factors are reported to relate to daytime sleepiness (arousal index = 60/h), but in an experiment that examined sleepiness and the relationship of the frequency of awakening once a minute, the arousal index was the strongest predictor of sleepiness in the daytime and subjective sleepiness, and there was no change in sleepiness in the daytime associated with awakening (arousal index = 6/h) once in 10 min [19, 20].

The criteria of respiratory event particularly hypopnea are changed every several years after the 2000s [21, 22]. Nevertheless, we used the decision method same as the same technique for PSG in this study from 2000 throughout. Hypopnea was defined as a 50 % reduction in air flow and/or respiratory effort, accompanied by oxygen desaturation of more than 3 %, despite of following arousal.

Because the AHI strongly correlated with arousal index (*r* = 0.881), 3%ODI (*r* = 0.966), and 4%ODI (*r* = 0.943) in this study, we performed a multivariate analysis with these factors separately. However, all of these factors were not extracted as an occurrence of accident factor.

We identified ESS and feeling drowsy more than sometimes during regular driving and working as risk factors related to automobile accidents due to falling asleep while driving. Accordingly, we coded the presence of accidents due to drowsy driving as positive and calculated an ROC curve for ESS to determine the sensitivity and specificity by ESS score. The specificity was 0.605 and sensitivity 0.642 at an ESS score of 11, and specificity was 0.895 and sensitivity 0.263 at an ESS score of 16. In this study, we used 11 points on the ESS as the cutoff value indicating symptoms of hypersomnia. Even though those cutoff values are acceptable for the risk of accidents caused by falling asleep while driving, it is difficult to use a sensitivity of 0.642 and specificity of 0.605 for screening. On the other hand, at 16 points on the ESS, a sensitivity of 0.263 was too low, but a specificity of 0.895 was high. It seems that the ESS of 16 points and more was an important means of making the OSAS patient aware of the risk of automobile accidents caused by falling asleep while driving. Therefore, it is important for medical professionals to educate subjects on the risk of accidents due to driving while drowsy as well as to provide information about treatments and their efficacy. Providing proper knowledge to subjects should reduce the accident risk [23, 24].

More importantly, it would be beneficial if our findings could help to motivate people to become advocates for their own treatment by evaluating sleepiness using their ESS score prior to driving.

It has been established that nasal continuous positive airway pressure, the first-line treatment for OSAS, can improve sleepiness and lower the automobile accident rate [25]. Though application of these findings to commercial drivers (vs. the general driving population) is not clear, it seems that early detection of OSAS as a preventive measure is important.

Future research should study cognitive functioning, work hours, the number accidents, and quality of life. In addition, mandatory use of specialized equipment to reduce accidents, including automatic emergency braking and automatic urgent brakes, should be considered.

## Conclusion

In this study, ESS and “frequency of feeling drowsy while regular driving and working” were the significant factors associated with automobile accidents caused by falling asleep while driving. Although the ESS is a subjective measure and may not be available for screening, the ESS is a simple and easy means, and an important means of making the OSAS patient aware of the risk of automobile accidents caused by falling asleep while driving.
